# Metabolic Molecule PLA2G2D Is a Potential Prognostic Biomarker Correlating With Immune Cell Infiltration and the Expression of Immune Checkpoint Genes in Cervical Squamous Cell Carcinoma

**DOI:** 10.3389/fonc.2021.755668

**Published:** 2021-10-18

**Authors:** Hong Liu, Ruiyi Xu, Chun Gao, Tong Zhu, Liting Liu, Yifan Yang, Haihong Zeng, Yafei Huang, Hui Wang

**Affiliations:** ^1^ Cancer Biology Research Center (Key Laboratory of the Ministry of Education), Tongji Hospital, Tongji Medical College, Huazhong University of Science and Technology, Wuhan, China; ^2^ Department of Gynecologic Oncology, Tongji Hospital, Tongji Medical College, Huazhong University of Science and Technology, Wuhan, China; ^3^ Department of Gynecologic Oncology, Women’s Hospital, Zhejiang University School of Medicine, Hangzhou, China; ^4^ Department of Pathogen Biology, School of Basic Medicine, Tongji Medical College, Huazhong University of Science and Technology, Wuhan, China

**Keywords:** cervical squamous cell carcinoma, PLA2G2D, tumor immune microenvironment, immune infiltration, metabolism, multiplex immunohistochemistry

## Abstract

Cervical squamous cell carcinoma (CSCC) is the major pathological type of cervical cancer (CC), the second most prevalent reproductive system malignant tumor threatening the health of women worldwide. The prognosis of CSCC patients is largely affected by the tumor immune microenvironment (TIME); however, the biomarker landscape related to the immune microenvironment of CSCC and patient prognosis is less characterized. Here, we analyzed RNA-seq data of CSCC patients from The Cancer Genome Atlas (TCGA) database by dividing it into high- and low-immune infiltration groups with the MCP-counter and ESTIMATE R packages. After combining weighted gene co-expression network analysis (WGCNA) and differentially expressed gene (DEG) analysis, we found that *PLA2G2D*, a metabolism-associated gene, is the top gene positively associated with immune infiltration and patient survival. This finding was validated using data from The Cancer Genome Characterization Initiative (CGCI) database and further confirmed by quantitative reverse transcription-polymerase chain reaction (qRT-PCR). Finally, multiplex immunohistochemistry (mIHC) was performed to confirm the differential infiltration of immune cells between *PLA2G2D*-high and *PLA2G2D*-low tumors at the protein level. Our results demonstrated that *PLA2G2D* expression was significantly correlated with the infiltration of immune cells, especially T cells and macrophages. More importantly, *PLA2G2D*-high tumors also exhibited higher infiltration of CD8^+^ T cells inside the tumor region than *PLA2G2D*-low tumors. In addition, PLA2G2D expression was found to be positively correlated with the expression of multiple immune checkpoint genes (ICPs). Moreover, based on other immunotherapy cohort data, *PLA2G2D* high expression is correlated with increased cytotoxicity and favorable response to immune checkpoint blockade (ICB) therapy. Hence, PLA2G2D could be a novel potential biomarker for immune cell infiltration, patient survival, and the response to ICB therapy in CSCC and may represent a promising target for the treatment of CSCC patients.

## Introduction

Cervical cancer (CC) is the fourth common malignant disease for female with an estimated 604,000 new cases and 342,000 deaths worldwide in 2020 (1). Cervical squamous cell carcinoma (CSCC) and adenocarcinoma are the most common pathological types accounting for approximately 70% and 25% of all CC, respectively (2). Among them, CSCC is mainly related to human papillomavirus (HPV) 16 subtype infection and viral gene integration, whereas adenocarcinoma is often complicated with HPV18 infection (3). Despite the substantial efforts being made in promoting HPV vaccination and early screening, the incidence of CC remains alarming in developing countries (2). In fact, CC is the most frequently diagnosed female cancer in 23 countries according to the latest report (2). Current therapies for CC including surgical treatment, chemotherapy, and radiotherapy have greatly improved the clinical outcome of CC; however, the therapeutic efficacy remains limited for patients with advanced and distant conditions, which estimated a median overall survival of 17 months and 5-year survival of 17% (4, 5). Therefore, there is an urgent need for developing novel therapeutic strategies that can effectively treat these patients (6, 7).

Immunotherapy is one of such strategies that has become a rapidly developing field for cancer treatment including cervical cancer. Through replenishing a sufficient number of expanded autologous T cells that can specifically kill cancer cells, adoptive cell transfer (ACT) has shown great promise in treating CC patients with advance diseases. Two major strategies of ACT, tumor infiltrating lymphocyte (TIL) and T-cell receptor-engineered T cell (TCR-T), were reported to have the objective response rate (ORR) of 44.4% and 50% (8, 9), respectively. Immune checkpoint inhibitors (ICBs), another type of immunotherapy that target programmed death-1(PD-1) receptor or ligand and cytotoxic T-lymphocyte-associated protein 4 (CTLA4), have achieved great success in various kinds of tumors. However, in several clinical trials for advanced cervical cancer patients, the response rate to ICBs was relatively low (10–13). Previous studies have shown that multiple factors are correlated with the efficacy of ICB therapy. Among them, more immune cell infiltration, especially the density and localization of CD8^+^ T cells in the tumor immune microenvironment (TIME) has been demonstrated to be correlated with a favorable response to ICBs in various cancer types (14, 15). However, the prognostic value and underlying molecular mechanism of immune infiltration in CC with or without immunotherapy remain less characterized.

Here, we utilized the RNA-seq data of CSCC patients from The Cancer Genome Atlas (TCGA) database for the sake of finding biomarkers related to prognosis and immune cell infiltration. To this end, we used two immune scoring algorithms to divide them into two groups with high- and low-immune infiltration. By applying weighted gene co-expression network analysis (WGCNA) and differentially expressed gene (DEG) analysis, phospholipase A_2_ Group IID (*PLA2G2D*), an immune- and metabolism-associated molecule, was identified to be the biomarker which is predictive for patient prognosis and immune cell infiltration of CC. Furthermore, five immune-related genes (i.e., *SLAMF6*, *SLAMF1*, *SH2D1A*, *TRAT1*, and *ZNF831*) were found to be co-expressed with *PLA2G2D*. In addition, *PLA2G2D* expression was also found to be positively correlated with the expression of multiple ICP genes. Finally, through a series of bioinformatics analysis and experimental verification approaches at both the transcriptional and protein levels, we proved that PLA2G2D could be a novel biomarker correlating with immune infiltration, especially CD8^+^ T cells, in CSCC.

## Materials and Methods

### Data Source and Processing

The Cancer Genome Atlas (TCGA) CSCC RNA-seq gene expression matrix based on Illumina platform and phenotype data were downloaded from UCSC Xena (https://xenabrowser.net/datapages/). Two hundred fifty CSCC patients were selected for subsequent analysis according to pathological diagnosis of squamous cell carcinoma. Two types of data including raw counts and fragments per kilo base per million mapped (FPKM) reads were applied. FPKM data were converted to transcripts per kilobase of per million mapped (TPM) data. In addition, the validation cohort dataset also based on Illumina high-throughput platform was downloaded from The Cancer Genome Characterization Initiative (CGCI) database (16). RNA-seq data from two immunotherapy cohorts for melanoma (17) and urothelial cancer (18) were downloaded from a public database. The gene names of all expression matrix were de-annotated through “ClusterProfiler” and “org.Hs.eg.db” package based on R language (V4.1.1) (19).

### Estimation of Tumor Immune Infiltration and Cytolytic Activity Score

The Microenvironment Cell Populations-counter (MCP-counter) algorithm was applied to score the level of immune cell infiltration in tumor, based on which samples were classified into high- and low-immune infiltration groups using the hierarchical clustering method (20). Alternatively, the level of immune and stromal fraction was scored by Estimation of Stromal Immune cells in MAlignant Tumor tissues using Expression data (ESTIMATE) based on log_2_-transformed TPM data, and samples were further divided into high- and low-immune infiltration groups equally with the cutoff score set at median (21). In addition, EPIC (22) and quanTIseq (23) algorithm were also utilized to calculate immune cell proportion through TPM expression matrix. CYT score was calculated by the log-transformed geometric mean of GZMA and PRF1 TPM value (24).

### Gene Screening by WGCNA

The log_2_-transformed TPM value expression matrix was put into WGCNA R package to select immune-associated genes (25). Firstly, 12,417 genes with coefficient of variation (CV) >0.5 were selected for further analysis by WGCNA R package. Of note, five samples were excluded for the abnormal height value in sample dendrogram analysis. The power of *β* value was set at 3 to construct the topological overlap matrix (TOM). Next, we set the minimum module gene size at 30 and generated 63 gene modules with different colors based on hierarchical clustering method, then merged multiple similar gene modules into one. Finally, the correlation between gene modules and traits was calculated to determine the most relevant module and WGCNA filtered genes were screened by setting the standard of module membership (MM) >0.8 and gene significance (GS) >0.5. Genes network was constructed by Cytoscape (V3.8.0) based on the genes with weight value >0.20 (26).

## Differentially Expressed Gene Analysis and Pathways Enrichment Analysis

In order to determine the DEGs between the high- and low-immune infiltration groups, the raw read count matrix of 250 CSCC patients from TCGA database was brought into DESeq2 (27). Genes with |Log_2_(FoldChange)| >1 and adjusted *P*-value <0.01 were considered as DEGs. ClusterProfiler R package was used to perform Gene Ontology (GO) term enrichment analysis for biological pathway. CGCI data matrix was imported into GSEA software (V4.1.0) for gene set enrichment analysis using the Hallmark gene sets database.

### Tissue Collection and Processing

Tumor samples from 18 CSCC patients were collected in Tongji Hospital, Tongji Medical College, Huazhong University of Science and Technology. No patient received radiotherapy and chemotherapy before tissue collection. Each tissue was divided into two parts for RNA extraction and formalin-fixed and paraffin-embedded (FFPE), respectively. FFPE tissues were cut into 4-μm-thick sections on slides. This research was approved by the Ethical Committee of Tongji Hospital, Tongji Medical College, Huazhong University of Science and Technology (approval number: TJ-IRB2021207).

### RNA Extraction and Quantitative Real-Time PCR

Total RNA was extracted and dissolved by RNA Isolation Kit (Vazyme, RC-112-01). cDNA library was obtained using a quantitative real-time reverse transcription-polymerase chain reaction (qRT-PCR) reagent kit (Vazyme, R223-01). The qRT-PCR reaction system contained 1 μg cDNA, 0.4 μl forward primer, 0.4 μl reverse primer, 8.2 μl H_2_O, and 10 μl 2× ChamQ Universal SYBR qPCR Master Mix (Vazyme, R223-01). GAPDH was set as internal control for gene quantification. The expression level of each gene was detected at least three times. The primer sequences are listed in **Supplementary Table 1**.

### Multiplex Immunohistochemistry

All FFPE slides were deparaffinized by dipping in xylene for 1 h and then rehydrated using the gradient ethanol method. After deparaffinization and rehydration, all slides were put into boiled AR6 retrieval solution for heat-induced epitope retrieval in a microwave for 15 min. Endogenous peroxidase was eliminated with 3% H_2_O_2_ for 15 min. Slides were cooled to room temperature followed by washing with 1× Tris-buffered saline-Tween 20 (TBST) buffer. Then, Opal 7-color manual IHC kit (Akoya Biosciences, NEL811001KT) was used to stain several markers in a single FFPE slide. To block non-specific protein binding sites, slides were incubated with blocking buffer (Antgene, ANT041) at room temperature for 10 min. Subsequently, FFPE tissue slides were incubated with primary antibody at 37°C for 30 min or 4°C overnight, HRP-labeled secondary antibody for 10 min, and Opal fluorescein for 10 min, successively. To stain four markers in a single slide, four rounds of the above staining procedure were performed with indicated primary antibody and matched Opal fluorescein pairs (**Supplementary Table 2**). The slides were always washed three times between each step with 1× TBST buffer. After four rounds, samples were stained for cellular DNA with 4′,6-diamidino-2-phenylindole (DAPI) (1:10, Servicebio, G1012-10ML) for 10 min followed by mounting with Fluoromount-G (SouthernBiotech, 0100-01) and preserved in the dark at 4°C. All slides were scanned by Verctra V3.0 System, and ×4 and ×20 objective lens were used to acquire low-power and high-power images, respectively. Images were analyzed with Phenochart V1.0 software and inForm V2.4 software for tissue component segmentation of tumor, stroma, and glass regions, respectively. To identify cell phenotypes, a threshold of fluorescein intensity was set for each marker. The ratio of the targeted cell counts and all cell counts in a certain region was used to calculate the percentage of immune cells in each region. The ratio of cell counts and area (cell/mm^2^) was utilized to reveal the density of a certain cell population.

### Survival Analysis and Statistical Analysis

Survival information of TCGA CSCC patients was downloaded from the UCSC Xena database. Patients who survived less than 30 days were excluded from this analysis. Kaplan–Meier survival analysis was applied to educe the correlation between 5-year overall survival and the expression level of indicated genes. Based on the relationship with survival time, survival status, and minus PLA2G2D TPM value, ROC curve analysis was applied by survivalROC R package. Statistical analysis was performed with R statistical package. Wilcoxon rank-sum test was employed for comparison between two groups. *P*-values <0.05 were considered significant: *P < 0.05, **P < 0.01, ***P < 0.001, and ****P < 0.0001.

## Results

### Classification of CSCC by Immune Cell Infiltration

**Figure 1:**
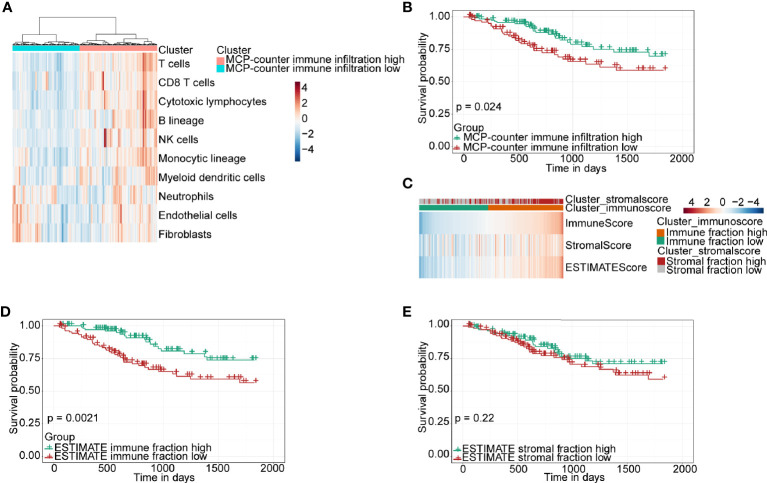
Immune classification and survival analysis of 250 CSCC patients selected from TCGA database. **(A)** Heatmap of TME composition as defined by the MCP-counter algorithm. **(B)** Kaplan–Meier survival curve for immune infiltration-high and infiltration-low groups determined by the MCP-counter. **(C)** Heatmap showing immune and stromal fraction by the ESTIMATE algorithm. **(D, E)** Kaplan–Meier survival curve for patients with high and low fractions of immune cells and stromal cells classified by the ESTIMATE algorithm.

The RNA-seq data and clinical information of a CSCC cohort including 250 patients were downloaded from TCGA database for bioinformatics analysis. Firstly, the MCP-counter, a widely used algorithm, was used to evaluate the level of immune infiltration for each sample, according to which CSCC patients were divided into high- and low-immune infiltration clusters based on the unsupervised stratification method (**Figure 1A**). Subsequently, the 5-year overall survival of the high- and low-immune infiltration groups was compared. The results demonstrated that the high-immune infiltration cluster determined by the MCP-counter had significantly higher survival than the low-immune infiltration cluster in CSCC (**Figure 1B**). Next, ESTIMATE, another algorithm which can similarly stratify CSCC patients into high- and low-immune fraction clusters by a median cutoff (**Figure 1C**), was chosen to verify this finding. Again, the high-immune infiltration cluster classified by ESTIMATE was found to have higher survival than the low-immune infiltration cluster in CSCC (**Figure 1D**). Of note, ESTIMATE can also classify CSCC patients into high- and low-stromal fraction clusters (**Figure 1C**). However, no difference was found between the two stromal fraction-stratified clusters in terms of patient survival (**Figure 1E**, *P* = 0.22). Together, our results demonstrated that immune infiltration, but not stromal fraction, was correlated with patient survival in CSCC. Moreover, the other two algorithms, EPIC and quanTIseq, were used to calculate several immune cell proportions compared with the MCP-counter and ESTIMATE. Moreover, the immune cell infiltration results of EPIC and quanTIseq were consistent with the MCP-counter and ESTIMATE (**Figure S1**).

### Construction of Weighted Gene Co-Expression Network and Genes Filtering Analysis

**Figure 2:**
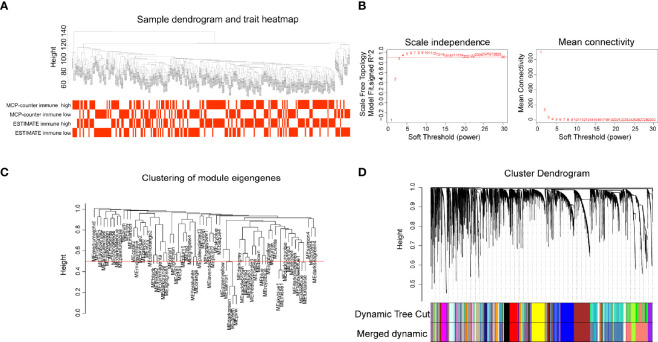
Gene modules construction by weighted gene co-expression network analysis (WGCNA). **(A)** Sample dendrogram and trait heatmap. **(B)** The relationship of soft threshold with scale independence and mean connectivity. **(C)** Clustering of module eigengenes, height set at 0.5 to merge modules. **(D)** Cluster dendrogram showing the hierarchical cluster tree for the identified co-expression gene modules with different colors.

CSCC RNA-seq data from TCGA was imported into WGCNA R package to identify gene modules containing similarly expressed genes, especially immune-related genes. Sample clustering and trait distribution are shown in **Figure 2A**. Power value was set at *β* = 3 to build a scale-free network (**Figure 2B**). Gene modules were calculated and the cutoff height was set at 0.5 to merge similar gene modules into one (**Figure 2C**). This analysis resulted in 63 merged dynamic gene modules with each module containing dozens to more than a thousand genes (**Figure 2D**, **Supplementary Table 3**).

**Figure 3:**
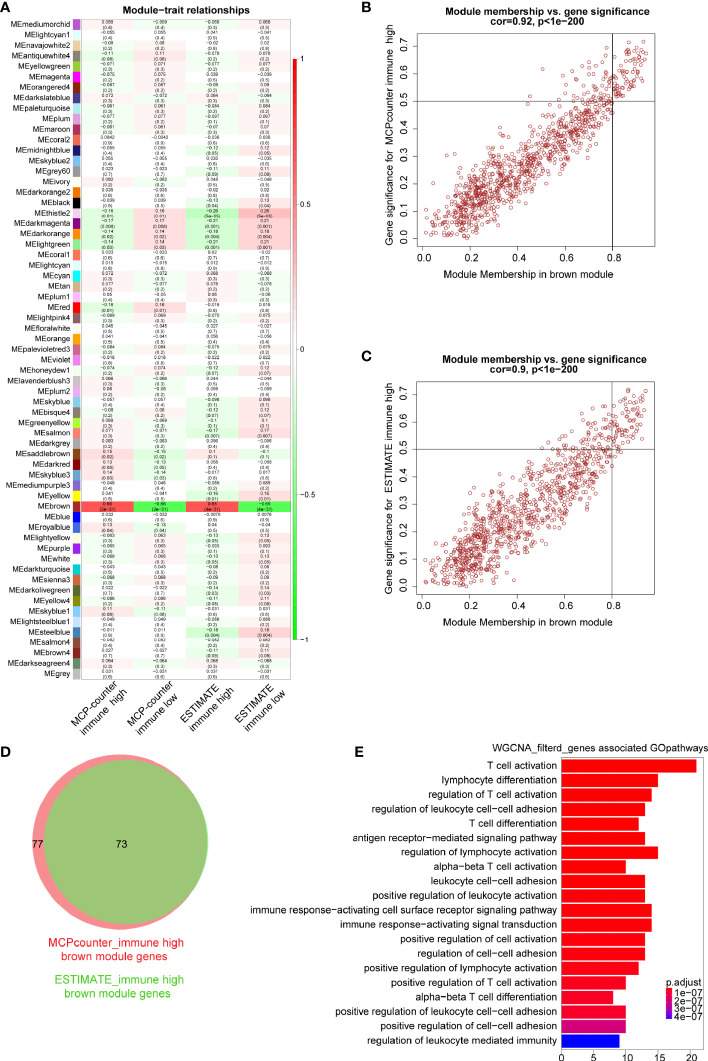
Key modules and genes identified based on module–trait relationship. **(A)** The relationship between co-expression modules and traits; each grid includes the degree of correlation and *P*-value. **(B, C)** Scatter plots showing the relationship of module membership (MM) in brown module, with gene significance (GS) for high-immune infiltration determined by the MCP-counter and ESTIMTE algorithms, for which MM >0.8 and GS >0.5 were set as gene filtered standard, respectively. **(D)** Venn plot showing common filtered genes identified in the high-immune infiltration groups determined by the MCP-counter and ESTIMATE. **(E)** GO analysis for WGCNA filtered genes in the biological process.

In order to identify immune-related modules and genes, we constructed the relationship between gene modules and immune infiltration traits (**Figure 3A**). We focused on the brown module which was most relevant to immune infiltration or immune fraction determined by the MCP-counter and ESTIMATE, respectively. To further identify genes in the brown module that are most correlated with the MCP-counter immune infiltration and ESTIMATE immune fraction, the screening criteria of MM >0.8 and GS >0.5 were applied, through which 77 genes (brown module *vs*. MCP-counter immune infiltration) and 73 genes (brown module *vs*. ESTIMATE immune fraction) were filtered (**Figures 3B, C**). Next, Venn plot was used to reveal the overlapped filtered genes of these two groups. As shown in **Figure 3D**, all 73 brown module genes contained in the ESTIMATE high-immune fraction group were found to be presented in the MCP-counter high-immune infiltration group as well. Finally, GO pathways enrichment analysis for the shared 73 WGCNA genes showed that these genes were mostly involved in immune-associated pathways, such as T-cell activation, lymphocyte differentiation, and regulation of T-cell activation (**Figure 3E**).

### *PLA2G2D* Is Positively Correlated With Immune infiltration and Patient Survival

**Figure 4:**
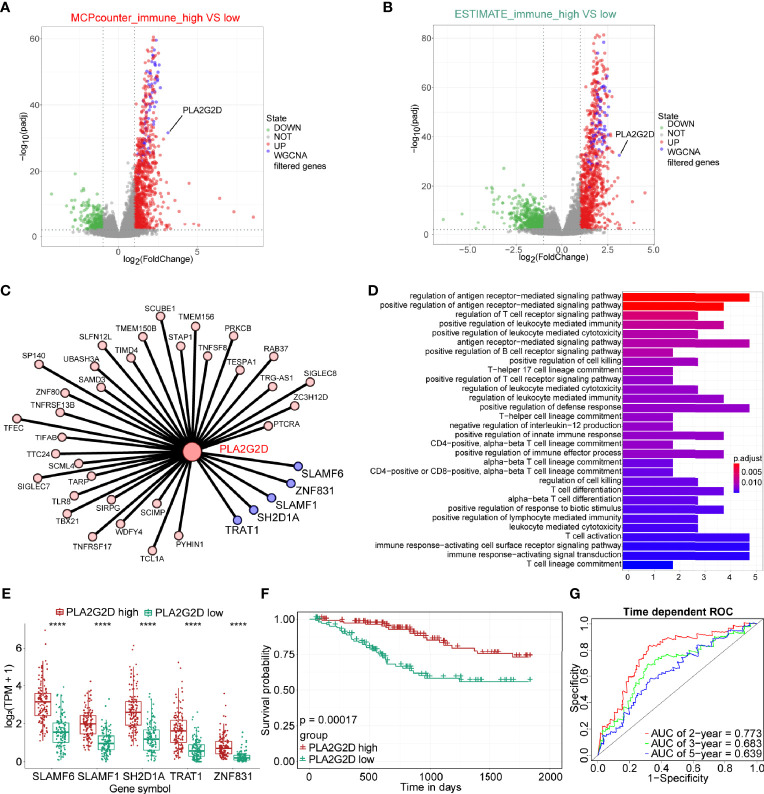
The expression of PLA2G2D is positively correlated with immune infiltration and patient survival. Volcano plot for DEGs identified for high- and low-immune infiltration groups based on the MCP-counter **(A)** and ESTIMATE **(B)**. The green and red dots represented the significantly downregulated and upregulated genes, respectively, and the gray dots represented the undifferentiated genes; the purple dots showed the WGCNA filtered genes, with |Log_2_(FoldChange)| >1 and adjusted *P*-value <0.01. **(C)** Cytoscape network plot showed the relationship between *PLA2G2D* and other co-expressed genes with weight value >0.2. The purple nodes showing the co-expressed genes with weight value >0.3. **(D)** GO pathways enrichment analysis for genes co-expressed with *PLA2G2D*. **(E)** Histogram showing the relationship between the expression levels of *PLA2G2D* and other five co-expressed genes, ****P < 0.0001. **(F)** Kaplan–Meier survival curve plot showing the relationship between PLA2G2D expression level and patient survival. **(G)** Time-dependent ROC curve analysis for minus *PLA2G2D* TPM value at 2-, 3-, and 5-year cutoffs.

To further explore hub genes from these 73 genes, DEG analysis was performed using DESeq2 R package. DEGs were determined between the high- and low-immune infiltration groups determined by the MCP-counter, through which 968 upregulated genes and 323 downregulated genes were found in the high- and low-immune infiltration groups, respectively (**Figure 4A**). Similarly, 1,060 upregulated genes and 650 downregulated genes were found in the high- and low-immune fraction groups determined by ESTIMATE, respectively (**Figure 4B**). Interestingly, all 73 WGCNA filtered genes were presented in these two upregulated gene sets. Notably, *PLA2G2D*, a metabolism-related gene, was identified as the most differentially expressed gene among the 73 genes, as determined by the log_2_ (FoldChange) value of these genes (**Table 1** and **Figures 4A, B**).

**Table 1:** Top 20 WGCNA filtered genes determined by DEG analysis of high- and low-immune infiltration groups classified by the MCP-counter and ESTIMATE algorithms.

Order	Genes	MCP-counter immune high *vs*. low		Genes	ESTIMATE immune high *vs*. low	
		Log2FoldChange	*P*adj		Log2FoldChange	*P*adj
1	*PLA2G2D*	3.120339747	2.46E-32	*PLA2G2D*	3.129951852	3.07E-33
2	*GZMK*	2.633961849	6.52E-46	*CRTAM*	2.594071877	3.75E-59
3	*TRAT1*	2.565309728	8.77E-53	*TRAT1*	2.543058375	7.56E-53
4	*CRTAM*	2.493534917	1.38E-50	*GZMK*	2.526206854	1.77E-41
5	*SCML4*	2.461018797	1.85E-48	*SH2D1A*	2.46209327	2.63E-65
6	*GPR174*	2.451822301	5.68E-48	*SCML4*	2.456859681	1.78E-49
7	*ZNF831*	2.379191341	4.28E-49	*LINC01857*	2.45160968	1.25E-36
8	*LINC00861*	2.373752175	1.36E-41	*SIRPG*	2.414521914	1.33E-64
9	*SLAMF6*	2.370123928	2.92E-60	*FCRL3*	2.36471631	1.57E-43
10	*GPR171*	2.345873252	6.25E-49	*TBX21*	2.359189375	9.61E-61
11	*SH2D1A*	2.329233049	1.14E-52	*SLAMF6*	2.334677345	2.09E-59
12	*LY9*	2.298409977	2.26E-47	*LY9*	2.327353913	1.71E-50
13	*SIRPG*	2.296040614	5.79E-53	*TLR8*	2.325276706	5.66E-45
14	*TBX21*	2.2919668	1.58E-53	*TIFAB*	2.310890798	6.39E-41
15	*FCRL6*	2.28625033	9.57E-45	*LINC00861*	2.299844149	8.68E-39
16	*TTC24*	2.274489399	3.53E-40	*CD3G*	2.277158384	2.71E-61
17	*CD3G*	2.254820652	2.70E-57	*SP140*	2.272328994	4.91E-79
18	*EOMES*	2.21785005	9.37E-36	*ZNF831*	2.271868811	1.21E-43
19	*FCRL3*	2.206529839	7.39E-35	*GPR174*	2.269780489	3.33E-39
20	*LINC01857*	2.184645997	2.81E-26	*LINC00426*	2.223943929	5.04E-61

Next, we sought to identify the genes that were co-expressed with *PLA2G2D*. The co-expression network between *PLA2G2D* and other genes was constructed by using the criteria of weight >0.20 (**Figure 4C** and **Table 2**). To gain insight into the function of these co-expressed genes, GO analysis of the biological process was conducted and the most co-expressed genes were enriched in immune-related pathways (**Figure 4D**). Of note, several genes with weight >0.3 including *SLAMF6*, *SLAMF1*, *SH2D1A*, *TRAT1*, and *ZNF831* were most co-expressed with *PLA2G2D* (**Figure 4E**). Importantly, Kaplan–Meier survival curve analysis showed that the expression level of *PLA2G2D* was positively correlated with patient survival (**Figure 4F**, *P* = 0.00017). Finally, ROC curve was constructed to demonstrate the prognostic ability of *PLA2G2D* in TCGA-CSCC cohort. The AUCs of PLA2G2D TPM minus value for 2, 3, and 5 years were 0.773, 0.683, and 0.639, respectively (**Figure 4G**).

**Table 2:** List of genes co-expressed with *PLA2G2D* with weight value >0.20.

*PLA2G2D* co-expressed genes	Weight	*PLA2G2D* co-expressed genes	Weight
SLAMF6	0.316522209	TLR8	0.224623497
SH2D1A	0.313611578	ZC3H12D	0.218210964
TRAT1	0.308061949	TIFAB	0.218179744
ZNF831	0.306000355	SIGLEC8	0.217411803
SLAMF1	0.300894868	SLFN12L	0.215513035
TESPA1	0.295561708	TTC24	0.214768858
PYHIN1	0.29293532	TNFRSF13B	0.213697367
WDFY4	0.279759397	TIMD4	0.211705633
UBASH3A	0.274771647	TCL1A	0.211489274
SAMD3	0.270901098	PTCRA	0.208689904
SCML4	0.270390467	TARP	0.208642553
PRKCB	0.26625189	TMEM156	0.207822287
SIRPG	0.262965895	ZNF80	0.206555702
TFEC	0.260430877	TMEM150B	0.20555541
TBX21	0.25827151	STAP1	0.204952535
SP140	0.256619486	RAB37	0.204841733
TRG-AS1	0.250638121	TNFRSF17	0.203896246
TNFSF8	0.246175611	SCUBE1	0.203302527
SCIMP	0.240444493	SIGLEC7	0.200210069

### Bioinformatic Validation Using Data From the CGCI Database and PCR Validation Using Tumor Tissues

**Figure 5:**
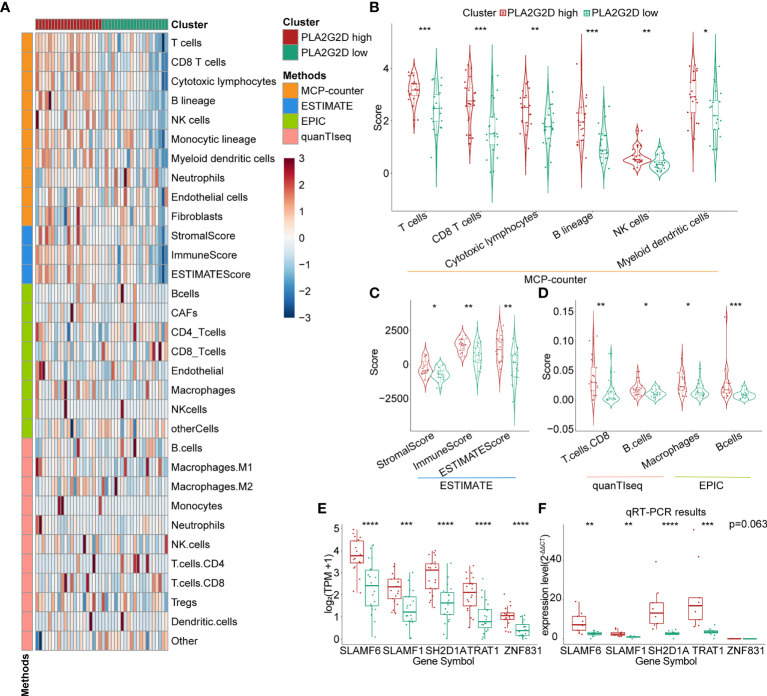
Further validation of the relationship between *PLA2G2D* expression and immune infiltration. **(A)** Heatmap for PLA2G2D high- and low-expression groups based on the MCP-counter, ESTIMATE, EPIC, and quanTIseq algorithms using data from the CGCI database. **(B)** Violin plot showing the differential scores of six immune cell types determined by the MCP-counter algorithm. **(C)** Violin plot showing the differential StromalScore, ImmuneScore, and ESTIMATEScore determined by the ESTIMATE algorithm. **(D)** Violin plot showing the differential immune cell types determined by quanTIseq and EPIC algorithms. **(E, F)** Histograms showing the relationship between the expression levels of PLA2G2D and WGCNA filtered five co-expressed genes using data from the CGCI database **(D)** and freshly isolated clinical samples **(E)**. *P < 0.05, **P < 0.01, ***P < 0.001, and ****P < 0.0001.

To validate the correlation between overexpressed *PLA2G2D* and more immune cell infiltration demonstrated in **Figure 1**, we analyzed RNA-seq data of CSCC from the CGCI database that was also sequenced on an Illumina high-throughput sequence platform. The immune characteristics of patients in the *PLA2G2D* high- and low-expression clusters are illustrated in **Figure 5A** by the analysis with the four algorithms. Overexpressed *PLA2G2D* was bound up with more immune cell infiltration such as T cells, B cells, NK cells, and myeloid dendritic cells (**Figure 5B**). Similarly, ImmuneScore, StromalScore, and ESTIMATEScore were higher in the *PLA2G2D* high-expression cluster than in the *PLA2G2D* low-expression cluster (**Figure 5C**). CD8^+^ T cells, macrophages, and B cells were also higher in the PLA2G2D-high group based on quanTIseq and EPIC algorithms (**Figure 5D**). GSEA analysis showed that the *PLA2G2D* high-expression cluster was enriched in several immune-associated pathways such as allograft rejection, interferon gamma response, interferon alpha response, complement, and oxidative phosphorylation pathways (**Figure S2A**), whereas *PLA2G2D* low-expression cluster was enriched in hypoxia, apical junction, glycolysis, mitotic spindle, and angiogenesis pathways (**Figure S2B**). We also validated whether *PLA2G2D* was co-expressed with other genes as indicated by the aforementioned WGCNA analysis. Our analysis demonstrated that the expression level of *PLA2G2D* was also positively associated with *SLAMF6*, *SLAMF1*, *SH2D1A*, *TRAT1*, and *ZNF831* when the CGCI database was used (**Figure 5E**). To further validate these results using fresh-isolated clinical samples, 18 cervical squamous carcinoma samples were collected and the corresponding clinical data are shown in **Table 3**. Total RNA per sample was isolated and qRT-PCR was performed to examine the gene expression levels of *PLA2G2D* and other five genes (**Table 4**). Our cohort was classified into *PLA2G2D* high- and low-expression groups according to the median expression level of *PLA2G2D*. Similarly, the expression levels of four immune-related genes were significantly higher in the *PLA2G2D* high-expression group, except for *ZNF831*, which could hardly be detected by qRT-PCR in both groups (**Figure 5F** and **Table 4**).

**Table 3:** Clinical characteristics of patients from our CSCC cohort.

Classification	Total	Low *PLA2G2D* expression	High *PLA2G2D* expression
Age			
<40	3	2	1
40–49	8	5	3
≥50	7	2	5
FIGO stage			
I–IIA	10	6	4
IIB–IV	8	3	5
Vascular invasion			
Positive	6	4	2
Negative	12	5	7
Lymphatic metastasis			
Positive	8	3	5
Negative	10	6	4

**Table 4:** qRT-PCR ΔCT value of *PLA2G2D* and other five co-expressed genes.

Samples	Genes						*PLA2G2D* group
	*SLAMF6*	*SLAMF1*	*SH2D1A*	*TRAT1*	*ZNF831*	*PLA2G2D*	
SCC11	16.24	16.25	16.25	15.5	20.23	20.06	Low
SCC5	10.59	11.81	10.48	10.78	19.92	15.8	Low
SCC4	10.24	12.03	10.47	9.93	19	14.61	Low
SCC17	11.46	11.97	10.33	10.05	21.07	13.19	Low
SCC6	9.5	11.68	9.89	9.58	18.09	13.15	Low
SCC10	10.55	11.59	10.23	9.28	19.01	12.99	Low
SCC9	9.88	10.95	9.57	9.57	19.72	12.62	Low
SCC1	9.75	11.23	9.34	8.78	18.81	12.39	Low
SCC7	10.15	11.59	10.04	9.81	17.72	12.09	Low
SCC2	10.44	11.47	9.3	8.97	19.58	11.87	High
SCC18	9.48	10.78	7.89	7.82	19.14	11.8	High
SCC15	10.08	10.74	8.79	8.85	17.65	11.69	High
SCC3	8.84	10.11	7.67	7.53	19.53	11.11	High
SCC16	8.77	9.89	8.64	8.32	18.3	11.1	High
SCC14	8.09	10.35	8.11	7.22	16.35	10.72	High
SCC13	8.45	9.15	7.41	7.39	18.16	10.23	High
SCC8	7.91	11.25	7.06	6.22	16.8	9.99	High
SCC12	7.36	9.31	6.31	5.81	17.32	9.54	High

### More Immune Cell Infiltration Determined by Multiplex Immunohistochemistry in *PLA2G2D* High-Expression Samples

**Figure 6:**
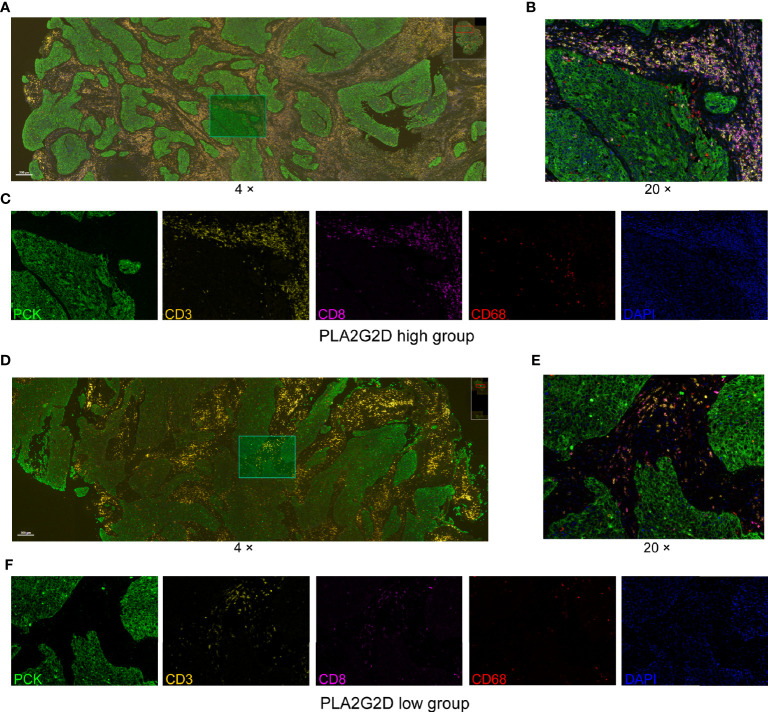
mIHC staining for CD3, CD8, CD68, PCK, and DAPI in cervical squamous cancer specimens with high **(A–C)** and low expression **(D–F)** of the *PLA2G2D* gene. **(A, D)** Merged mIHC images at low magnification (×4). **(B, E)** Merged mIHC images at high magnification (×20) indicating the same filed in **(A, D)**. **(C, F)** Single spectral images indicating the same filed in **(B, E)**.

**Figure 7:**
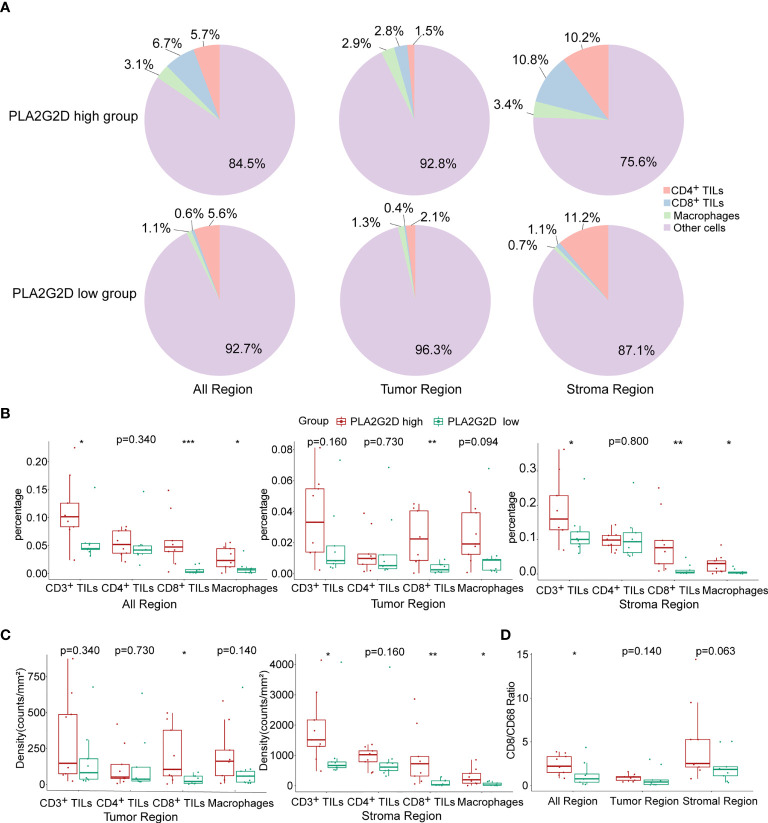
Statistical analysis for mIHC staining. **(A)** Pie plots showing the composition of immune cells within different regions in *PLA2G2D* high- and low-expression groups. **(B)** Histograms showing the comparison of the percentage of individual immune cell populations between *PLA2G2D* high- and low- expression groups in different regions. **(C)** Histograms showing the comparison of the density of individual immune cell populations between *PLA2G2D* high- and low-expression groups in different regions. **(D)** Histogram showing the comparison of the CD8/CD68 ratio between *PLA2G2D* high- and low-expression groups in different regions. *P < 0.05, **P < 0.01, and ***P < 0.001.

Next, the multiplex immunohistochemistry (mIHC) method was performed to validate whether immune cell infiltration was correlated with *PLA2G2*D expression in our cohort of 18 CSCC clinical samples. As our aforementioned bioinformatic analysis indicated that T cells and macrophages were the most prevalent cells among the infiltrated immune cells positively correlated with *PLA2G2D* expression, and previous studies have shown that the number and percentage of T cells and macrophages were more than the other kind of immune cells in cervical cancer (28, 29), we chose these two cells along with tumor cells to determine the percentage and density of individual immune cell populations in each slide. For this purpose, the mIHC panel (**Supplementary Table 2**) was designed to simultaneously stain several markers including CD3 (for all T cells), CD8 (for cytotoxic T cells), CD68 (for macrophages), PCK (for tumor cells), and DAPI (for nucleic identification). Of note, CD3^+^CD8^−^ TILs were defined as CD4^+^ TILs. Our results indicated that CD3^+^ T cells, including both CD4^+^ and CD8^+^ TILs, and CD68^+^ macrophages were more abundant in the *PLA2G2D*-high group than in the *PLA2G2D*-low group, as shown by representative merged images at low magnification (**Figures 6A, D**) and high magnification (**Figures 6B, E**) and by single spectrum images for each marker (**Figures 6C, F**). After comparing *PLA2G2D*-high and *PLA2G2D*-low groups for the compositions of individual cell populations in different regions (**Figure 7A**) and the percentages of individual cell populations in each sample (**Figure S3**), we found that the percentage values of CD3^+^ TILs, CD8^+^ TILs, and macrophages in the stromal region and all regions of the *PLA2G2D* high-expression group were significantly higher compared with that of the *PLA2G2D* low-expression group (**Figure 7B**). Importantly, in the tumor region, CD8^+^ TILs were the only cell population that were more frequently observed in the *PLA2G2D* high-expression group (**Figure 7B**).

Next, cell density (cell counts/mm^2^ area) was analyzed in the tumor and stromal region, respectively. Similar to the comparison of cell percentage, cell densities of CD3^+^ TILs, CD8^+^ TILs, and macrophages were significantly higher in the stromal region of the *PLA2G2D* high-expression group than those of the *PLA2G2D* low-expression group. Moreover, in the tumor region, only CD8^+^ TIL density in the *PLA2G2D* high-expression group was higher compared with that in the *PLA2G2D* low-expression group (**Figure 7C**).

Tumor-resident macrophages were previously considered as a major cell population in suppressing the function of cytotoxic T cells and were reported to be associated with poor prognosis of cancer (30). Therefore, we assessed the CD8/CD68 ratio between these two groups. The ratio was higher in all areas in the *PLA2G2D* high-expression group (**Figure 7D**).

### Positive Correlation Between the Expression of *PLA2G2D* and ICPs

**Figure 8:**
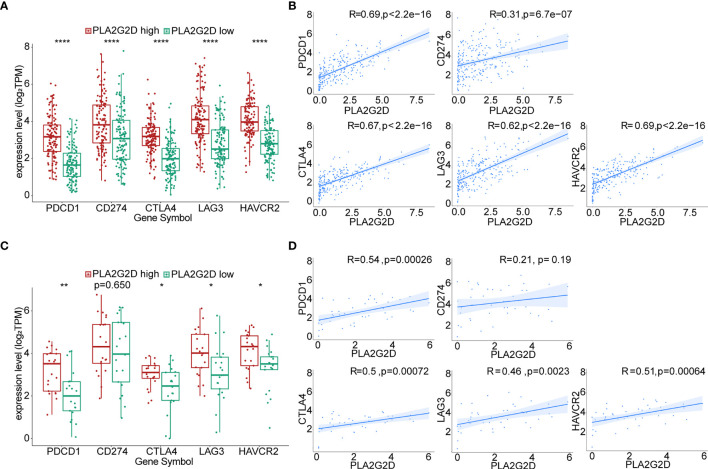
Correlation of the expression level of *PLA2G2D* and ICPs. Histograms showing the comparison of ICP expression level between *PLA2G2D*-high and *PLA2G2D*-low groups in TCGA **(A)** and CGCI database **(C)**. Pearson correlation analysis for the expression level of *PLA2G2D* and ICPs in TCGA **(B)** and CGCI **(D)** database. *P < 0.05, **P < 0.01, and ****P < 0.0001.

The expression of immune checkpoint genes [e.g., *PDCD1* (*PD1*), *CD274* (*PDL1*), *CTLA4*, *LAG3*, and *HAVCR2* (*TIM3*)] has been utilized in predicting the response of patients to ICB therapy in a variety of cancers including CC. To evaluate the possible relationship between the expression of *PLA2G2D* and these ICPs, correlation analysis was performed using transcriptomic data from TCGA database. Our analysis indicated that the expression of ICP genes was significantly higher in the *PLA2G2D* high-expression group compared with that in the *PLA2G2D* low-expression group. Through Pearson correlation analysis, we found that these common ICP genes were positively correlated with *PLA2G2D* expression (**Figures 8A, C**). Similar findings were noted when using the CGCI validation cohort except for *CD274*, the expression of which was comparable in the *PLA2G2D* low-expression and high-expression groups and was not correlated with the expression of *PLA2G2D*. This discrepancy may be attributable to the small sample size in this cohort (**Figures 8B, D**).

### Positive Correlation Between the Expression of *PLA2G2D* and the Response to ICB Therapy in Immunotherapy Cohorts

**Figure 9:**
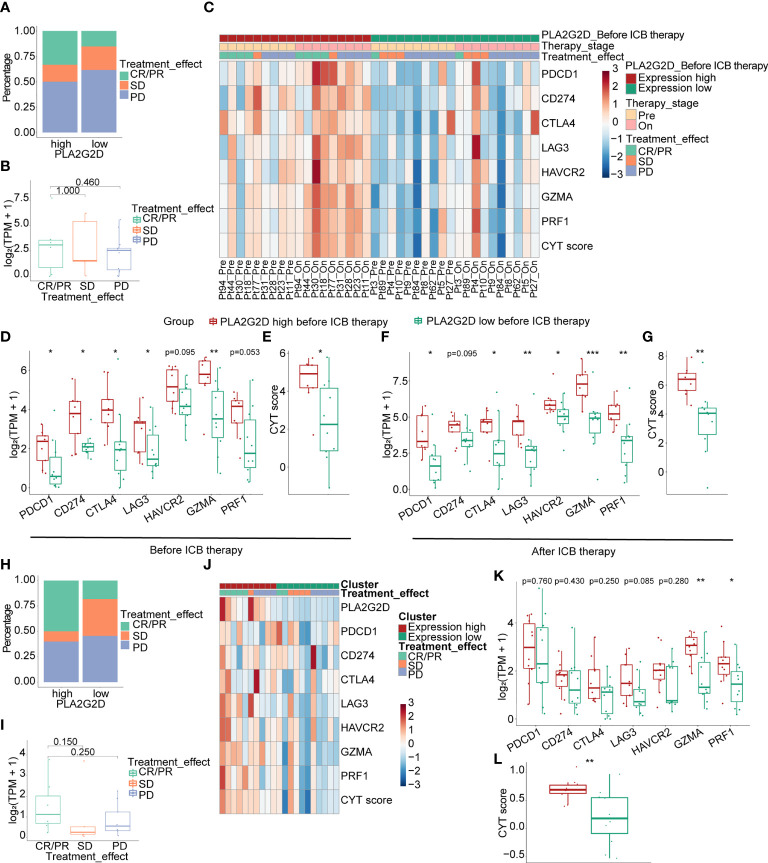
Correlation of the expression level of *PLA2G2D* and response to ICB treatment in melanoma **(A–G)** and urothelial carcinoma immunotherapy cohorts **(H–L)**. **(A, B)** Bar and boxplot for the relationship between *PLA2G2D* expression and response to ICB treatment in melanoma cohort. **(C)** Heatmap of the relationship of ICPs, cytotoxic genes, and CYT score with different *PLA2G2D* expression levels and response to treatment in paired pre- and on-treatment patients. Boxplots showing the differential expression of ICPs, cytotoxic genes, and CYT scores between patients with different *PLA2G2D* expression levels before ICB therapy **(D, E)** and after ICB therapy **(F, G)**. **(H, I)** Bar and boxplot for the relationship between *PLA2G2D* expression and response to ICB treatment in urothelial carcinoma cohort. **(J)** Heatmap of the relationship of ICPs, cytotoxic genes, and CYT score with different *PLA2G2D* expression levels. **(K, L)** Boxplots showing the differential expression of ICPs, cytotoxic genes, and CYT score between patients with different *PLA2G2D* expression levels. *P < 0.05, **P < 0.01, and ***P < 0.001.

ICB therapy has been performed in patients with advanced cervical cancer, but transcriptional data for these cohorts are not accessible. To validate the relationship between *PLA2G2D* and the response to ICB treatment, we analyzed the transcriptome data from immunotherapy cohorts of melanoma and urothelial carcinoma. In the melanoma anti-PD1 therapy cohort, we selected patients who have not previously received immunotherapy for enrollment analysis and divided them based on *PLA2G2D* expression at pretreatment stage. The percentage of compete response or partial response (CR/PR) was higher in *PLA2G2D*-high patients (**Figure 9A**). PLA2G2D expression level of CR/PR patients also showed a higher tendency compared with stable disease (SD) and progressive disease (PD) patients; however, these differences failed to reach statistical significance (**Figure 9B**). In this cohort, 9 and 10 pairs of pre- and on-treatment patients were assigned to the PLA2G2D-high and PLA2G2D-low cluster, respectively. Before ICB therapy, *PLA2G2D*-high patients show higher ICP expression and CYT score (**Figures 9C–E**). Interestingly, after ICB therapy, *PLA2G2D*-high patients also have higher ICP expression and CYT score (**Figures 9C, F, G**). Importantly, compared with pretreatment patients, on-treatment patients have stronger cytotoxicity and higher ICP expression after anti-PD-1 therapy regardless of *PLA2G2D*-high or *PLA2G2D*-low groups (**Figure 9C**). Furthermore, in the urothelial carcinoma anti-PDL1 therapy cohort, the percentage of CR/PR patients was higher in *PLA2G2D*-high patients, which is similar to that in melanoma immunotherapy cohort (**Figure 9H**). CR/PR patients showed a higher tendency of *PLA2G2D* expression level than SD and PD patients (**Figure 9I**). Unfortunately, only pretreatment transcriptome data are available for this cohort, thus preventing us from performing similar analysis for on-treatment data. Nevertheless, *PLA2G2D*-high expression was also accompanied by high expression of ICPs despite no statistical significance (**Figures 9J, K**), while expression level of cytotoxicity markers (*GZMA* and *PRF1*) and the CYT score were significantly higher in the *PLA2G2D*-high group (**Figures 9J–L**).

## Discussion

The tumor microenvironment (TME) is a complex milieu that comprises diverse cell populations including malignant cells, immune cells, stromal cells, and other cell types (31). Each cell population can impact others through releasing soluble molecules and/or by direct cell–cell interaction. As a crucial part of the tumor microenvironment, TIME plays a pivotal role in tumorigenesis and the clinical outcomes of cancer patients (32). Accumulating lines of evidence have suggested that a certain type of TIME characterized by more immune cell infiltration is indicative of a better prognosis and a favorable response to ICBs (15, 33). Furthermore, several factors from tumor cells themselves, immune cells, and stromal cells were reported to be implicated in shaping the landscape of TIME (34–36). However, for CSCC, the connection between TIME (especially immune infiltration) and cancer prognosis and the possible underpinning molecular mechanism are far from elucidated. This limitation greatly prevents the identification of new therapeutic targets that can be utilized to improve patient prognosis through transforming the TIME with poor-immune infiltration to high-immune infiltration. Therefore, this study was designed to address this issue. Through bioinformatics analyses, we identified a key immune- and metabolism-associated molecule PLA2G2D that was significantly related to better prognosis of CSCC patients and more immune cell infiltration.

Metabolism-associated molecules are critical components of TIME (37). Metabolic reprograming of tumor cells may promote tumor growth and metastasis directly or indirectly through influencing the functions of a variety of immune cells, especially T cells (38–40), and the shape of TIME. Phospholipase A2 (PLA_2_) proteins are a group of lipid metabolism-associated molecules that can catalyze the hydrolysis of the sn-2 position of membrane glycerophospholipids to release unsaturated fatty acid and lysophospholipid, thereby playing a pivotal role in multiple biochemical processes including inflammatory response. While both cytosolic and secreted forms of PLA_2_ have been identified, more than one-third of the PLA_2_ enzymes belong to the secreted PLA_2_ (sPLA_2_) family, which have both pro- and anti-inflammatory functions as a result of the production of diverse lipid mediators (41, 42). As a member of sPLA_2_, group IID PLA_2_ (encoded by *PLA2G2D*), is broadly expressed in human body, such as the spleen, lymph nodes, squamous epithelium, and colorectal cancer tissue (43–45). In the current investigation, PLA2G2D was also found to be expressed in CSCC specimens at the transcriptional level (**Figure 5E**). Previous studies have also shown that PLA2G2D was preferentially expressed in lymphoid tissue-resident dendritic cells and macrophages and implicated in anti-inflammation response in an array of inflammation-related conditions including contact hypersensitivity (43), viral infection-associated inflammation (46), experimental encephalomyelitis and colitis (47), and contact dermatitis and psoriasis (48). The role of *Pla2g2d* in cancer has been investigated as well. Using both *Pla2g2d-*deficient and *Pla2g2d*-transgenic mouse models, Miki et al. found that *Pla2g2d* could facilitate the development of chemical-induced skin cancer accompanied by macrophage polarization toward M2 phenotype and decreased number of cytotoxic T cells (48). The cancer-promoting effect of PLA2G2D has also been suggested in human. Recently, *PLA2G2D* was identified as one of the high-risk genes for colorectal and rectum adenocarcinoma that were negatively correlated with patient survival. However, *PLA2G2D* expression was also found to be positively correlated with immune infiltration and better prognosis in head and neck squamous cell carcinoma and breast cancer in human (49, 50), which is consistent with our current findings in CSCC. The opposite effects of PLA2G2D in different cancer types may result from the distinct microenvironments and different downstream lipid mediators presented in each cancer type, which in turn result in either an elevated or dampened inflammatory responses, thereby leading to the distinct clinical outcomes reported in these investigations (48).

In this study, the relationship between *PLA2G2D* overexpression and more immune cell infiltration in CSCC was validated through two approaches. Firstly, another dataset from the CGCI database was used for validation at the transcriptional level. Secondly, mIHC, a multispectral microscopy technique that can reveal the TIME profile on FFPE slides, was applied to determine the major immune cell populations infiltrated in tumor including CD4^+^ T cells, CD8^+^ T cells, and macrophages, which were subsequently used to confirm their relationship with *PLA2G2D* expression. Our results demonstrated that *PLA2G2D* high-expressed samples had more immune cells infiltrated in the stroma region. More importantly, CD8^+^ TILs were the only cell population that were more frequently found inside the tumor region in *PLA2G2D* high-expressed samples, suggesting that PLA2G2D could participate in the recruitment of CD8^+^ TILs into the cancer nests, either directly or indirectly. The presence of tumor-associated macrophages is often associated with tumorigenesis, immune suppression, and poor prognosis of patients (35, 51). Indeed, macrophages can directly suppress CD8^+^ T-cell proliferation *in vitro* (30, 52–54). Therefore, we compared the CD8/CD68 ratio between *PLA2G2D*-high and *PLA2G2D*-low samples. Again, a significantly higher ratio of CD8/CD68 was found in *PLA2G2D*-high samples compared with that in *PLA2G2D*-low samples. Taken together, these results not only confirmed the aforementioned relationship between immune infiltration and *PLA2G2D* expression, but also revealed the spatial characteristics of immune infiltration in *PLA2G2D*-high samples.

The mechanism by which PLA2G2D affects immune infiltration in CSCC is currently unclear, and lipid mediators downstream of PLA2G2D might contribute. For example, the enzyme activity of PLA2G2D could result in the production of leukotriene B4, a potent chemotactic molecule with the capacity to recruit neutrophils, monocytes/macrophages, CD8^+^ cytotoxic T lymphocyte, and Th17 cells (55). However, molecules other than these lipid mediators may be involved as well. To explore the possible candidates, genes that co-expressed with *PLA2G2D* were determined by WGCNA analysis, which resulted in the identification of the top 5 genes, namely, *SLAMF6*, *SLAMF1*, *SH2D1A*, *TRAT1*, and *ZNF831.* These genes were successfully validated using another dataset from the CGCI database and confirmed by qRT-PCR using fresh-isolated CSCC samples, with the exception of *ZNF831*, the expression of which was extremely low and no statistical significance was found between *PLA2G2D*-high and *PLA2G2D*-low samples. It is noteworthy that these five genes are all associated with immunity. *SLAMF1 (CD150)* and *SLAMF6* belong to the signaling lymphocytic activation molecule (SLAM) family and express on several immune cells including T cells, B cells, and NK cells (56). Previous studies have shown that the expressions of *SLAMF1* in cervical cancer and *SLAMF6* in liver cancer were closely related to tumor infiltration of immune cells and patient prognosis (57, 58). The *SH2D1A* gene encodes SH2 domain-containing protein 1A, which is often known as SLAM-associated protein. SLAM can transduce the tyrosine signaling pathway to promote interferon-γ production in the presence of SH2D1A (56, 59). *TRAT1* encodes the tripartite motif (TRIM) protein which is essential for T-cell activation and positively correlated with the survival of patients with metastatic melanoma (59). *ZNF831* is a transcription factor gene and was also significantly correlated with immune cell infiltration in triple negative breast cancer (60). Together, in light of the role of the aforementioned co-expressed genes in various immune-related pathways, PLA2G2D may interact with these co-expressed genes indirectly or directly, thereby influencing immune cell infiltration.

Immune cell infiltration, along with PDL1 expression and tumor mutation burden (TMB), has been frequently used as independent biomarkers in predicting the response of patients to ICB therapy in multiple cancer types (61). Therefore, the positive correlation between immune cell infiltration and the expression of *PLA2G2D* in CSCC identified in this study indicates that *PLA2G2D* expression may also represent another predictive biomarker for ICB in CSCC. In support of this notion, we found that the levels of ICP genes, another independent biomarker for ICB therapy, were markedly higher in the *PLA2G2D* high-expression group than those in the *PLA2G2D* low-expression group at the transcriptional level. Furthermore, a positive correlation between the expression of *PLA2G2D* and ICP genes was noted in both TCGA and CGCI cohorts. Finally, we directly examined the predictive value of *PLA2G2D* expression for patient response to ICB therapy in melanoma and urothelial carcinoma immunotherapy cohorts, given the lack of transcriptional data in CSCC immunotherapy cohorts. Before ICB therapy, *PLA2G2D*-high patients have higher ICP expression and CYT score. After ICB therapy, the expression level of ICPs and CYT score were further increased compared with the pretreatment stage, as indicated by paired analysis. Hence, *PLA2G2D*-high patients have higher cytotoxicity and favorable response to ICB treatment. Taken together, *PLA2G2D* expression may represent a novel biomarker with a better predictive power for ICB therapy.

In conclusion, through integrating bioinformatics and experimental verification, we demonstrated that the metabolic molecule PLA2G2D was positively correlated with immune infiltration and patient prognosis in CSCC, suggesting that PLA2G2D could be a novel prognosis biomarker for CSCC patients. Furthermore, PLA2G2D might be a promising biomarker for the evaluation of immune infiltration situation across different patients and represent another independent predictive biomarker for ICB therapy in CSCC. However, the underpinning mechanism regarding how PLA2G2D works in TME is unclear and warrants further investigations.

## Data Availability Statement

The datasets presented in this study can be found in online repositories. The names of the repository/repositories and accession number(s) can be found in the article/**Supplementary Material**.

## Ethics Statement

The studies involving human participants were reviewed and approved by the Ethical Committee of Tongji Hospital, Tongji Medical College, Huazhong University of Science and Technology (approval number: TJ-IRB2021207). The patients/participants provided their written informed consent to participate in this study.

## Author Contributions

HW, YH, HL, and RX conceived and designed the study. HL did bioinformatics analysis and wrote the manuscript. HL and RX did the PCR and mIHC experiments and analyzed the data. CG designed the mIHC panel. HL, CG, TZ, LL, YY, and HZ handled the clinical samples and collected clinical data. HW and YH had supervised this project and contributed to writing and revision of the manuscript. All authors contributed to the article and approved the submitted version.

## Funding

This study was supported by grants from the National Natural Science Foundation of China (NSFC No. 81772786, No. 81830074) and the Research Funds from Tongji Hospital (No. 20185BJRC004, No. 2019BJRC008).

## Conflict of Interest

The authors declare that the research was conducted in the absence of any commercial or financial relationships that could be construed as a potential conflict of interest.

## Publisher’s Note

All claims expressed in this article are solely those of the authors and do not necessarily represent those of their affiliated organizations, or those of the publisher, the editors and the reviewers. Any product that may be evaluated in this article, or claim that may be made by its manufacturer, is not guaranteed or endorsed by the publisher.

## Supplementary Material

Supplementary Figure 1 Similarity of MCP-counter, ESTIMATE, EPIC and quanTIseq in characterizing immune infiltrations. Heatmap and boxplot showing the similarity between MCP-counter, EPIC and quanTIseq **(A, C)**. Heatmap and boxplot showing the similarity between ESTIMATE, EPIC and quanTIseq **(B, D)**. Pie plots showing the percentage of several kinds of immune cells based on EPIC **(E)** and quanTIseq **(F)** algorithms. **P* < 0.05, ***P* < 0.01, ****P* < 0.001, and *****P* < 0.0001.

Supplementary Figure 2 GSEA analysis for RNA-seq data from the CGCI database. **(A)** Several pathways enriched in *PLA2G2D* high-expression cluster. **(B)** Several pathways enriched in *PLA2G2D* low-expression cluster.

Supplementary Figure 3 Frequencies of the major cell populations for each sample calculated by mIHC method. Composition of different kind of cells for each sample in all region **(A)**, stromal region **(B)** and tumor region **(C)**.

## References

[1] Sung H Ferlay J Siegel RL Laversanne M Soerjomataram I Jemal A. Global Cancer Statistics 2020: GLOBOCAN Estimates of Incidence and Mortality Worldwide for 36 Cancers in 185 Countries. CA Cancer J Clin (2021) 71:209–49. doi: 10.3322/caac.21660 33538338

[2] Cohen PA Jhingran A Oaknin A Denny L. Cervical Cancer. Lancet (2019) 393:169–82. doi: 10.1016/S0140-6736(18)32470-X 30638582

[3] Hu Z Zhu D Wang W Li W Jia W Zeng X. Genome-Wide Profiling of HPV Integration in Cervical Cancer Identifies Clustered Genomic Hot Spots and a Potential Microhomology-Mediated Integration Mechanism. Nat Genet (2015) 47:158–63. doi: 10.1038/ng.3178 25581428

[4] Van Meir H Kenter GG Burggraaf J Kroep Jr Welters MJ Melief CJ. The Need for Improvement of the Treatment of Advanced and Metastatic Cervical Cancer, the Rationale for Combined Chemo-Immunotherapy. Anti-Cancer Agents in Medicinal Chemistry. Anticancer Agents Med Chem (2014) 14:190–203. doi: 10.2174/18715206113136660372 24237223

[5] National Cancer Institute. Cancer Stat Facts: Cervical Cancer. Cancer Statistics. (2021). Available at https://seer.cancer.gov/statfacts/html/cervix.html [Accessed October 9, 2021]

[6] Tewari KS Sill MW Long HJ 3rd Penson RT Huang H Ramondetta LM. Improved Survival With Bevacizumab in Advanced Cervical Cancer. N Engl J Med (2014) 370:734–43. doi: 10.1056/NEJMoa1309748 PMC4010094 24552320

[7] Tewari KS Monk BJ. New Strategies in Advanced Cervical Cancer: From Angiogenesis Blockade to Immunotherapy. Clin Cancer Res (2014) 20:5349–58. doi: 10.1158/1078-0432.CCR-14-1099 25104084

[8] Nagarsheth NB Norberg SM Sinkoe AL Adhikary S Meyer TJ Lack JB. TCR-Engineered T Cells Targeting E7 for Patients With Metastatic HPV-Associated Epithelial Cancers. Nat Med (2021) 27:419–25. doi: 10.1038/s41591-020-01225-1 PMC9620481 33558725

[9] Jazaeri AA Zsiros E Amaria RN Artz AS Edwards RP Wenham RM. Safety and Efficacy of Adoptive Cell Transfer Using Autologous Tumor Infiltrating Lymphocytes (LN-145) for Treatment of Recurrent, Metastatic, or Persistent Cervical Carcinoma. J Clin Oncol (2019) 37:2538–8. doi: 10.1200/JCO.2019.37.15_suppl.2538

[10] Lheureux S Butler MO Clarke B Cristea MC Martin LP Tonkin K. Association of Ipilimumab With Safety and Antitumor Activity in Women With Metastatic or Recurrent Human Papillomavirus-Related Cervical Carcinoma. JAMA Oncol (2018) 4:e173776. doi: 10.1001/jamaoncol.2017.3776 29145543 PMC6145732

[11] Petitprez F Meylan M De Reynies A Sautes-Fridman C Fridman WH. The Tumor Microenvironment in the Response to Immune Checkpoint Blockade Therapies. Front Immunol (2020) 11:784. doi: 10.3389/fimmu.2020.00784 32457745 PMC7221158

[12] Chung HC Ros W Delord JP Perets R Italiano A Shapira-Frommer. Efficacy and Safety of Pembrolizumab in Previously Treated Advanced Cervical Cancer: Results From the Phase II KEYNOTE-158 Study. J Clin Oncol (2019) 37:1470–8. doi: 10.1200/JCO.18.01265 30943124

[13] Frenel JS Le Tourneau C O'neil B Ott PA Piha-Paul SA Gomez-Roca C. Safety and Efficacy of Pembrolizumab in Advanced, Programmed Death Ligand 1-Positive Cervical Cancer: Results From the Phase Ib KEYNOTE-028 Trial. J Clin Oncol (2017) 35:4035–41. doi: 10.1200/JCO.2017.74.5471 29095678

[14] Tumeh PC Harview CL Yearley JH Shintaku IP Taylor EJ Robert L. PD-1 Blockade Induces Responses by Inhibiting Adaptive Immune Resistance. Nature (2014) 515:568–71. doi: 10.1038/nature13954 PMC4246418 25428505

[15] Herbst RS Soria JC Kowanetz M Fine GD Hamid O Gordon MS. Predictive Correlates of Response to the Anti-PD-L1 Antibody MPDL3280A in Cancer Patients. Nature (2014) 515:563–7. doi: 10.1038/nature14011 PMC4836193 25428504

[16] Gagliardi A Porter VL Zong Z Bowlby R Titmuss E Namirembe C. Analysis of Ugandan Cervical Carcinomas Identifies Human Papillomavirus Clade-Specific Epigenome and Transcriptome Landscapes. Nat Genet (2020) 52:800–10. doi: 10.1038/s41588-020-0673-7 PMC7498180 32747824

[17] Riaz N Havel JJ Makarov V Desrichard A Urba WJ Sims JS. Tumor and Microenvironment Evolution During Immunotherapy With Nivolumab. Cell (2017) 171:934–49.e916. doi: 10.1016/j.cell.2017.09.028 29033130 PMC5685550

[18] Snyder A Nathanson T Funt SA Ahuja A Buros Novik J Hellmann MD. Contribution of Systemic and Somatic Factors to Clinical Response and Resistance to PD-L1 Blockade in Urothelial Cancer: An Exploratory Multi-Omic Analysis. PLoS Med (2017) 14:e1002309. doi: 10.1371/journal.pmed.1002309 28552987 PMC5446110

[19] Yu G Wang LG Han Y He QY. Clusterprofiler: An R Package for Comparing Biological Themes Among Gene Clusters. OMICS (2012) 16:284–7. doi: 10.1089/omi.2011.0118 PMC3339379 22455463

[20] Becht E Giraldo NA Lacroix L Buttard B Elarouci N Petitprez F. Estimating the Population Abundance of Tissue-Infiltrating Immune and Stromal Cell Populations Using Gene Expression. Genome Biol (2016) 17:218. doi: 10.1186/s13059-016-1070-5 27765066 PMC5073889

[21] Yoshihara K Shahmoradgoli M Martinez E Vegesna R Kim H Torres-Garcia W. Inferring Tumour Purity and Stromal and Immune Cell Admixture From Expression Data. Nat Commun (2013) 4:2612. doi: 10.1038/ncomms3612 24113773 PMC3826632

[22] Racle J De Jonge K Baumgaertner P Speiser DE Gfeller D. Simultaneous Enumeration of Cancer and Immune Cell Types From Bulk Tumor Gene Expression Data. Elife (2017) 6:e26476. doi: 10.7554/eLife.26476 29130882 PMC5718706

[23] Finotello F Mayer C Plattner C Laschober G Rieder D Hackl H. Molecular and Pharmacological Modulators of the Tumor Immune Contexture Revealed by Deconvolution of RNA-Seq Data. Genome Med (2019) 11:34. doi: 10.1186/s13073-019-0638-6 31126321 PMC6534875

[24] Rooney MS Shukla SA Wu CJ Getz G Hacohen N. Molecular and Genetic Properties of Tumors Associated With Local Immune Cytolytic Activity. Cell (2015) 160:48–61. doi: 10.1016/j.cell.2014.12.033 25594174 PMC4856474

[25] Langfelder P Horvath S. WGCNA: An R Package for Weighted Correlation Network Analysis. BMC Bioinf (2008) 9:559. doi: 10.1186/1471-2105-9-559 PMC2631488 19114008

[26] Shannon P Markiel A Ozier O Baliga NS Wang JT Ramage D. Cytoscape: A Software Environment for Integrated Models of Biomolecular Interaction Networks. Genome Res (2003) 13:2498–504. doi: 10.1101/gr.1239303 PMC403769 14597658

[27] Love MI Huber W Anders S. Moderated Estimation of Fold Change and Dispersion for RNA-Seq Data With Deseq2. Genome Biol (2014) 15:550. doi: 10.1186/s13059-014-0550-8 25516281 PMC4302049

[28] Zou R Gu R Yu X Hu Y Yu J Xue X. Characteristics of Infiltrating Immune Cells and a Predictive Immune Model for Cervical Cancer. J Cancer (2021) 12:3501–14. doi: 10.7150/jca.55970 PMC8120169 33995627

[29] Wang J Li Z Gao A Wen Q Sun Y. The Prognostic Landscape of Tumor-Infiltrating Immune Cells in Cervical Cancer. BioMed Pharmacother (2019) 120:109444. doi: 10.1016/j.biopha.2019.109444 31562978

[30] Ruffell B Coussens LM. Macrophages and Therapeutic Resistance in Cancer. Cancer Cell (2015) 27:462–72. doi: 10.1016/j.ccell.2015.02.015 PMC4400235 25858805

[31] Lei X Lei Y Li JK Du WX Li RG Yang J. Immune Cells Within the Tumor Microenvironment: Biological Functions and Roles in Cancer Immunotherapy. Cancer Lett (2020) 470:126–33. doi: 10.1016/j.canlet.2019.11.009 31730903

[32] Su D Wu G Xiong R Sun X Xu M Mei Y. Tumor Immune Microenvironment Characteristics and Their Prognostic Value in Non-Small-Cell Lung Cancer. Front Oncol (2021) 11:634059. doi: 10.3389/fonc.2021.634059 33747957 PMC7966704

[33] Binnewies M Roberts EW Kersten K Chan V Fearon DF Merad M. Understanding the Tumor Immune Microenvironment (TIME) for Effective Therapy. Nat Med (2018) 24:541–50. doi: 10.1038/s41591-018-0014-x PMC5998822 29686425

[34] Li J Byrne KT Yan F Yamazoe T Chen Z Baslan T. Tumor Cell-Intrinsic Factors Underlie Heterogeneity of Immune Cell Infiltration and Response to Immunotherapy. Immunity (2018) 49:178–93.e177. doi: 10.1016/j.immuni.2018.06.006 29958801 PMC6707727

[35] Denardo DG Ruffell B. Macrophages as Regulators of Tumour Immunity and Immunotherapy. Nat Rev Immunol (2019) 19:369–82. doi: 10.1038/s41577-019-0127-6 PMC7339861 30718830

[36] Khalili JS Liu S Rodriguez-Cruz TG Whittington M Wardell S Liu C. Oncogenic BRAF(V600E) Promotes Stromal Cell-Mediated Immunosuppression via Induction of Interleukin-1 in Melanoma. Clin Cancer Res (2012) 18:5329–40. doi: 10.1158/1078-0432.CCR-12-1632 PMC3463754 22850568

[37] Cassim S Pouyssegur J. Tumor Microenvironment: A Metabolic Player That Shapes the Immune Response. Int J Mol Sci (2019) 21:157. doi: 10.3390/ijms21010157 PMC6982275 31881671

[38] Kolb D Kolishetti N Surnar B Sarkar S Guin S Shah AS. Metabolic Modulation of the Tumor Microenvironment Leads to Multiple Checkpoint Inhibition and Immune Cell Infiltration. ACS Nano (2020) 14:11055–66. doi: 10.1021/acsnano.9b10037 32706241

[39] Leone RD Zhao L Englert JM Sun IM Oh MH Sun IH. Glutamine Blockade Induces Divergent Metabolic Programs to Overcome Tumor Immune Evasion. Science (2019) 366:1013–21. doi: 10.1126/science.aav2588 PMC7023461 31699883

[40] Kishton RJ Sukumar M Restifo NP. Metabolic Regulation of T Cell Longevity and Function in Tumor Immunotherapy. Cell Metab (2017) 26:94–109. doi: 10.1016/j.cmet.2017.06.016 28683298 PMC5543711

[41] Lindbom J Ljungman AG Lindahl M Tagesson C. Increased Gene Expression of Novel Cytosolic and Secretory Phospholipase A(2) Types in Human Airway Epithelial Cells Induced by Tumor Necrosis Factor-Alpha and IFN-Gamma. J Interferon Cytokine Res (2002) 22:947–55. doi: 10.1089/10799900260286650 12396716

[42] Lambeau G Lazdunski M. Receptors for a Growing Family of Secreted Phospholipases A2. Trends Pharmacol Sci (1999) 20:162–70. doi: 10.1016/S0165-6147(99)01300-0 10322502

[43] Miki Y Yamamoto K Taketomi Y Sato H Shimo K Kobayashi T. Lymphoid Tissue Phospholipase A2 Group IID Resolves Contact Hypersensitivity by Driving Antiinflammatory Lipid Mediators. J Exp Med (2013) 210:1217–34. doi: 10.1084/jem.20121887 PMC3674707 23690440

[44] Mounier CM Wendum D Greenspan E Flejou JF Rosenberg DW Lambeau G. Distinct Expression Pattern of the Full Set of Secreted Phospholipases A2 in Human Colorectal Adenocarcinomas: Spla2-III as a Biomarker Candidate. Br J Cancer (2008) 98:587–95. doi: 10.1038/sj.bjc.6604184 PMC2243149 18212756

[45] Haas U Podda M Behne M Gurrieri S Alonso A Fürstenberger G. Characterization and Differentiation-Dependent Regulation of Secreted Phospholipases A2 in Human Keratinocytes and in Healthy and Psoriatic Human Skin. J Invest Dermatol (2005) 124:204–11. doi: 10.1111/j.0022-202X.2004.23513.x 15654975

[46] Vijay R Hua X Meyerholz DK Miki Y Yamamoto K Gelb M. Critical Role of Phospholipase A2 Group IID in Age-Related Susceptibility to Severe Acute Respiratory Syndrome-CoV Infection. J Exp Med (2015) 212:1851–68. doi: 10.1084/jem.20150632 PMC4612096 26392224

[47] Von Allmen CE Schmitz N Bauer M Hinton HJ Kurrer MO Buser RB. Secretory Phospholipase A2-IID Is an Effector Molecule of CD4+CD25+ Regulatory T Cells. Proc Natl Acad Sci USA (2009) 106:11673–8. doi: 10.1073/pnas.0812569106 PMC2710677 19564598

[48] Miki Y Kidoguchi Y Sato M Taketomi Y Taya C Muramatsu K. Dual Roles of Group IID Phospholipase A2 in Inflammation and Cancer. J Biol Chem (2016) 291:15588–601. doi: 10.1074/jbc.M116.734624 PMC4957044 27226632

[49] Ye Z Zou S Niu Z Xu Z Hu Y. A Novel Risk Model Based on Lipid Metabolism-Associated Genes Predicts Prognosis and Indicates Immune Microenvironment in Breast Cancer. Front Cell Dev Biol (2021) 9:691676. doi: 10.3389/fcell.2021.691676 34195202 PMC8236894

[50] Xiong Y Si Y Feng Y Zhuo S Cui B Zhang Z. Prognostic Value of Lipid Metabolism-Related Genes in Head and Neck Squamous Cell Carcinoma. Immun Inflamm Dis (2021) 9:196–209. doi: 10.1002/iid3.379 33277966 PMC7860527

[51] Mantovani A Marchesi F Malesci A Laghi L Allavena P. Tumour-Associated Macrophages as Treatment Targets in Oncology. Nat Rev Clin Oncol (2017) 14:399–416. doi: 10.1038/nrclinonc.2016.217 28117416 PMC5480600

[52] Doedens AL Stockmann C Rubinstein MP Liao D Zhang N Denardo DG. Macrophage Expression of Hypoxia-Inducible Factor-1 Alpha Suppresses T-Cell Function and Promotes Tumor Progression. Cancer Res (2010) 70:7465–75. doi: 10.1158/0008-5472.CAN-10-1439 PMC2948598 20841473

[53] Denardo DG Brennan DJ Rexhepaj E Ruffell B Shiao SL Madden SF. Leukocyte Complexity Predicts Breast Cancer Survival and Functionally Regulates Response to Chemotherapy. Cancer Discov (2011) 1:54–67. doi: 10.1158/2159-8274.CD-10-0028 22039576 PMC3203524

[54] Ruffell B Chang-Strachan D Chan V Rosenbusch A Ho CM Pryer N. Macrophage IL-10 Blocks CD8+ T Cell-Dependent Responses to Chemotherapy by Suppressing IL-12 Expression in Intratumoral Dendritic Cells. Cancer Cell (2014) 26:623–37. doi: 10.1016/j.ccell.2014.09.006 PMC4254570 25446896

[55] Jiang X Nicolls MR Tian W Rockson SG. Lymphatic Dysfunction, Leukotrienes, and Lymphedema. Annu Rev Physiol (2018) 80:49–70. doi: 10.1146/annurev-physiol-022516-034008 29029593 PMC6434710

[56] Veillette A Latour S. The SLAM Family of Immune-Cell Receptors. Curr Opin Immunol (2003) 15:277–85. doi: 10.1016/S0952-7915(03)00041-4 12787752

[57] Chen Q Qiu B Zeng X Hu L Huang D Chen K. Identification of a Tumor Microenvironment-Related Gene Signature to Improve the Prediction of Cervical Cancer Prognosis. Cancer Cell Int (2021) 21:182. doi: 10.1186/s12935-021-01867-2 33766042 PMC7992856

[58] Liu X Niu X Qiu Z. A Five-Gene Signature Based on Stromal/Immune Scores in the Tumor Microenvironment and Its Clinical Implications for Liver Cancer. DNA Cell Biol (2020) 39:1621–38. doi: 10.1089/dna.2020.5512 32758021

[59] Bogunovic D O'neill DW Belitskaya-Levy I Vacic V Yu YL Adams S. Immune Profile and Mitotic Index of Metastatic Melanoma Lesions Enhance Clinical Staging in Predicting Patient Survival. Proc Natl Acad Sci U S A (2009) 106:20429–34. doi: 10.1073/pnas.0905139106 PMC2787158 19915147

[60] He Y Jiang Z Chen C Wang X. Classification of Triple-Negative Breast Cancers Based on Immunogenomic Profiling. J Exp Clin Cancer Res (2018) 37:327. doi: 10.1186/s13046-018-1002-1 30594216 PMC6310928

[61] Gibney GT Weiner LM Atkins MB. Predictive Biomarkers for Checkpoint Inhibitor-Based Immunotherapy. Lancet Oncol (2016) 17:e542–51. doi: 10.1016/S1470-2045(16)30406-5 PMC5702534 27924752

