# Supplementary figures and images for: Metabolic Molecule PLA2G2D Is a Potential Prognostic Biomarker Correlating With Immune Cell Infiltration and the Expression of Immune Checkpoint Genes in Cervical Squamous Cell Carcinoma
Source: Front Oncol. 2021 Oct 18;11:755668. doi: 10.3389/fonc.2021.755668 (PMC8558485; doi:10.3389/fonc.2021.755668)

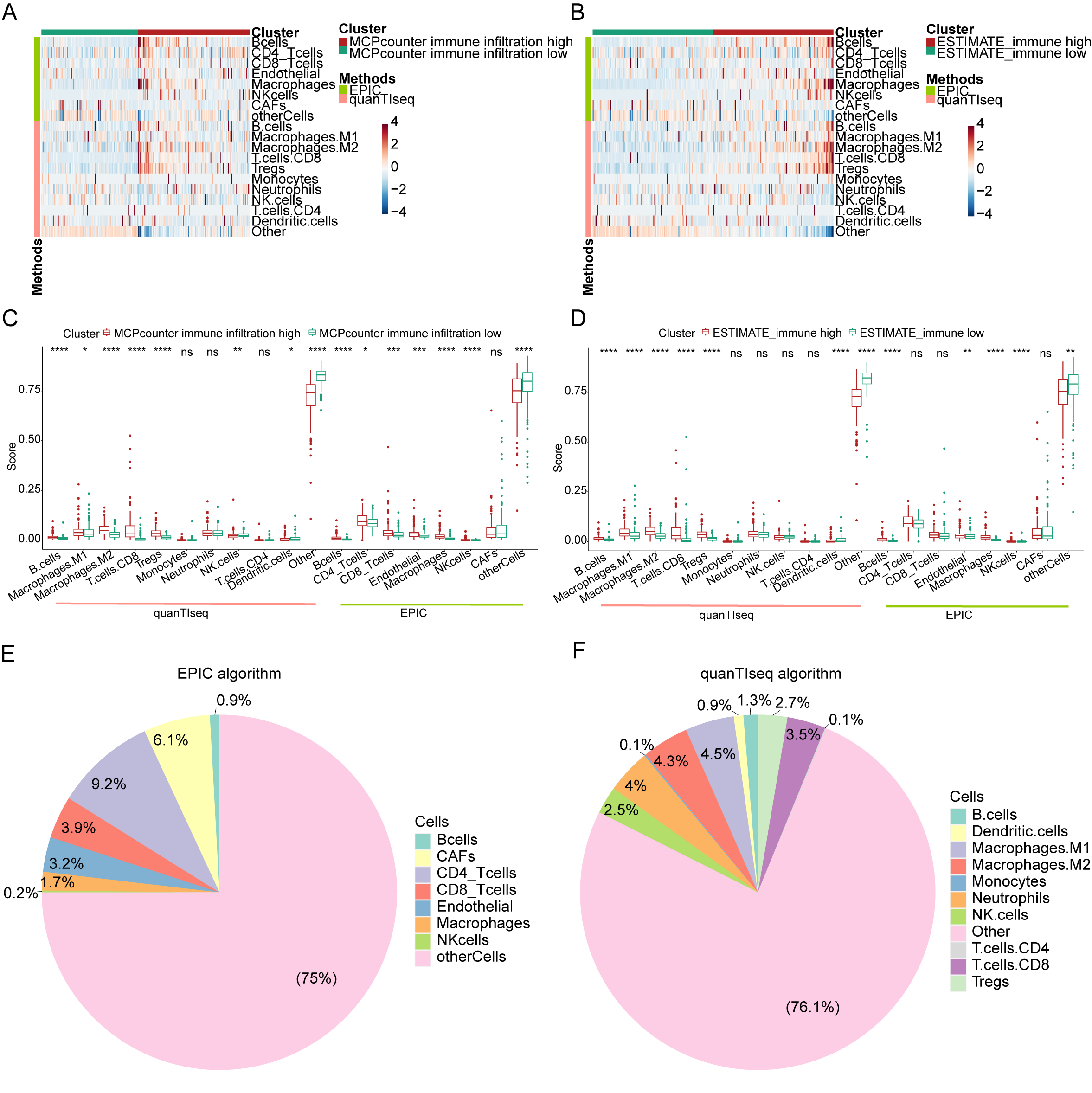

Supplement: Supplementary Figure 1 — Similarity of MCP-counter, ESTIMATE, EPIC and quanTIseq in characterizing immune infiltrations. Heatmap and boxplot showing the similarity between MCP-counter, EPIC and quanTIseq (A, C). Heatmap and boxplot showing the similarity between ESTIMATE, EPIC and quanTIseq (B, D). Pie plots showing the percentage of several kinds of immune cells based on EPIC (E) and quanTIseq (F) algorithms. *P < 0.05, **P < 0.01, ***P < 0.001, and ****P < 0.0001. [file Image_1.tif]

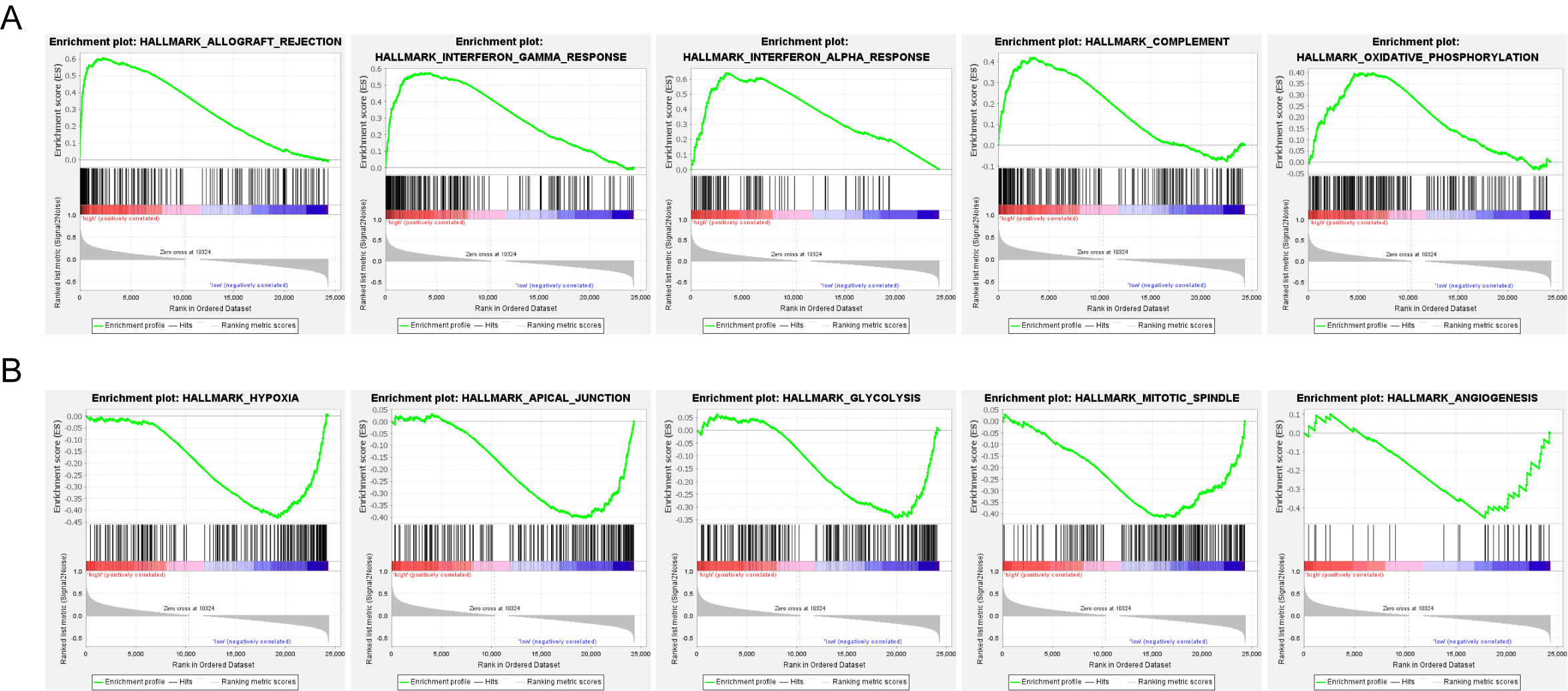

Supplement: Supplementary Figure 2 — GSEA analysis for RNA-seq data from the CGCI database. (A) Several pathways enriched in PLA2G2D high-expression cluster. (B) Several pathways enriched in PLA2G2D low-expression cluster. [file Image_2.tif]

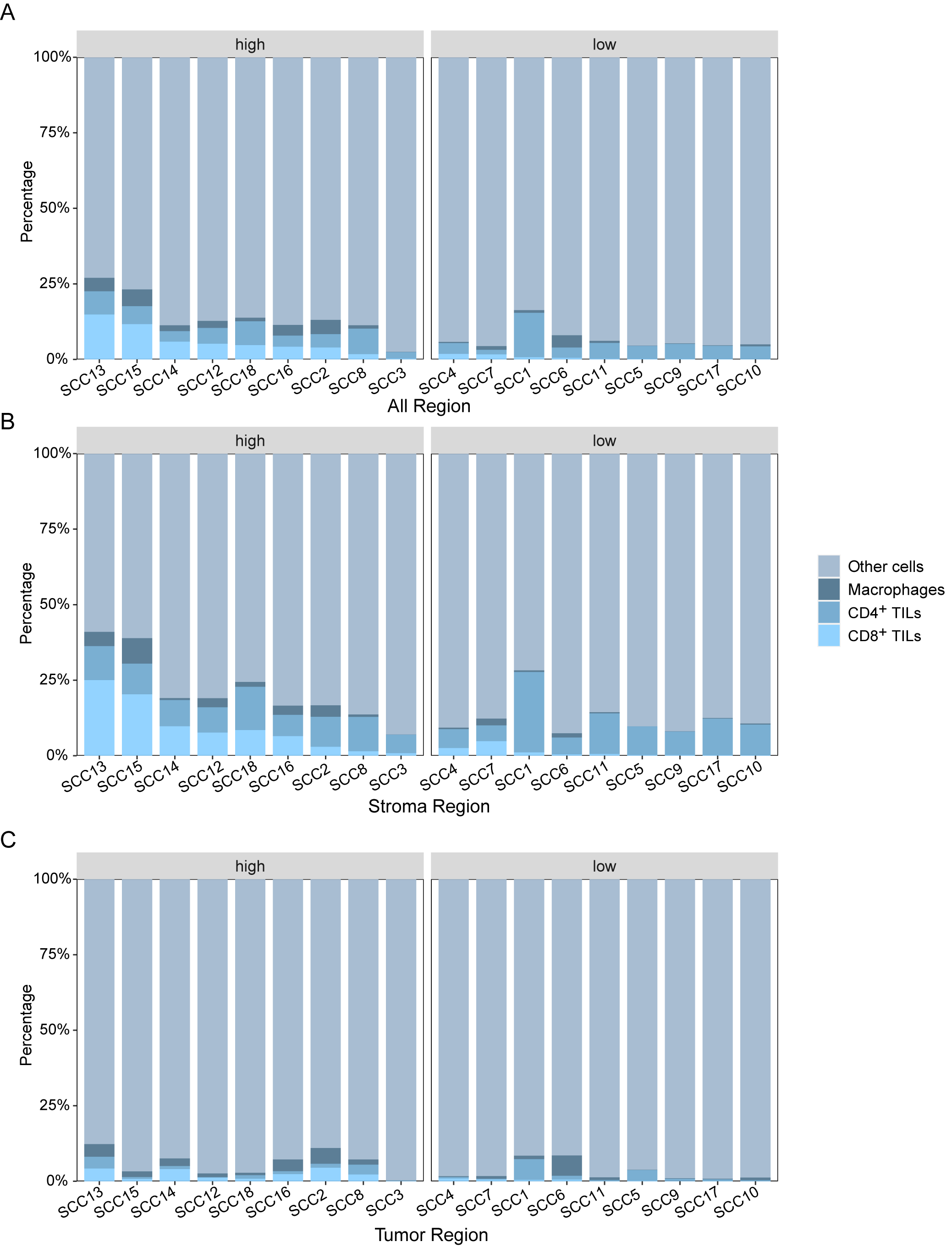

Supplement: Supplementary Figure 3 — Frequencies of the major cell populations for each sample calculated by mIHC method. Composition of different kind of cells for each sample in all region (A), stromal region (B) and tumor region (C). [file Image_3.tif]
