# Supplementary material for: Metabolic Molecule PLA2G2D Is a Potential Prognostic Biomarker Correlating With Immune Cell Infiltration and the Expression of Immune Checkpoint Genes in Cervical Squamous Cell Carcinoma
Source: Front Oncol. 2021 Oct 18;11:755668. doi: 10.3389/fonc.2021.755668 (PMC8558485; doi:10.3389/fonc.2021.755668)
Supplement: Supplementary file 4 [file Table_1.docx]

Supplementary Table 1. Specific primers for *SLAMF6*, *SLAMF1*, *SH2D1A,* *TRAT1*, *ZNF831*, *PLA2GA2D* and *GAPDH*.

| Genes | Prime Sequences |
| --- | --- |
| *SLAMF6* | Forward: 5'-GAGTCCGCAAGGAACCTAGAG-3'  Reverse: 5'-TCCCTGTTTGAATGAGTGACTGA-3' |
| *SLAMF1* | Forward: 5'-GGAGAACAGTGTCGAGAACAAA-3'  Reverse: 5'-CGTATCCCCAGGGTGAGATTC-3' |
| *SH2D1A* | Forward: 5'-AGGCGTGTACTGCCTATGTG-3'  Reverse: 5'-TGCAGAGGTATTACAATGCCTTG-3' |
| *TRAT1* | Forward: 5'-CTGGGGACTTCTAGCATTGTTG-3'  Reverse: 5'-CTCTGGTCGGGCTTTCATTTG-3' |
| *ZNF831* | Forward: 5'-TGTGGCTACAAAAATGAGACATTC-3'  Reverse: 5'-CCACAATGCTGGAAAACCTTGA-3' |
| *PLA2G2D* | Forward: 5'-ACTTTTCCCAGGGGAACATCC-3'  Reverse: 5'-GCAGTCGCTTCTGGTAGGTG-3' |
| *GAPDH* | Forward: 5'-CTGGGCTACACTGAGCACC-3'  Reverse: 5'-AAGTGGTCGTTGAGGGCAATG-3' |
