# Supplementary material for: Metabolic Molecule PLA2G2D Is a Potential Prognostic Biomarker Correlating With Immune Cell Infiltration and the Expression of Immune Checkpoint Genes in Cervical Squamous Cell Carcinoma
Source: Front Oncol. 2021 Oct 18;11:755668. doi: 10.3389/fonc.2021.755668 (PMC8558485; doi:10.3389/fonc.2021.755668)
Supplement: Supplementary file 5 [file Table_2.docx]

Supplementary Table 2. Primary antibodies and matched Opal fluorescein for mIHC analysis.

|  | | | Primary antibodies | | | | | | Opal  fluorescein |
| --- | --- | --- | --- | --- | --- | --- | --- | --- | --- |
| Antigen  specificity | Source | Clone number | | Catalog# | Dilution rate | Staining  temperature | Staining  times | Company |  |
| CD3 | Rabbit | SP7 | | Kit-0003 | 1:10 | 37℃ | 30 min | MXB biotechnologies | Opal570 |
| CD8 | Mouse | C8/144B | | MAB-0021 | 1:10 | 4℃ | overnight | MXB biotechnologies | Opal650 |
| CD68 | Rabbit | D4B9C | | 79594T | 1:8000 | 37℃ | 30 min | Cell Signaling Technology | Opal690 |
| PCK | Mouse | AE1/AE3 | | Kit-0003 | 1:10 | 37℃ | 30 min | MXB biotechnologies | Opal520 |
