# Supplementary material for: Metabolic Molecule PLA2G2D Is a Potential Prognostic Biomarker Correlating With Immune Cell Infiltration and the Expression of Immune Checkpoint Genes in Cervical Squamous Cell Carcinoma
Source: Front Oncol. 2021 Oct 18;11:755668. doi: 10.3389/fonc.2021.755668 (PMC8558485; doi:10.3389/fonc.2021.755668)
Supplement: Supplementary file 6 [file Table_3.docx]

Supplementary Table 3. WGCNA gene modules and the number of genes in each module.

| Merged Colors | Gene counts | Merged Colors | Gene counts |
| --- | --- | --- | --- |
| antiquewhite4 | 45 | maroon | 52 |
| bisque4 | 64 | mediumorchid | 43 |
| black | 431 | mediumpurple3 | 79 |
| blue | 1172 | midnightblue | 177 |
| brown | 1000 | navajowhite2 | 55 |
| brown4 | 66 | orange | 116 |
| coral1 | 46 | orangered4 | 80 |
| coral2 | 44 | paleturquoise | 93 |
| cyan | 194 | palevioletred3 | 55 |
| darkgrey | 122 | plum | 34 |
| darkmagenta | 91 | plum1 | 84 |
| darkolivegreen | 92 | plum2 | 62 |
| darkorange | 114 | purple | 249 |
| darkorange2 | 67 | red | 459 |
| darkred | 132 | royalblue | 134 |
| darkseagreen4 | 47 | saddlebrown | 97 |
| darkslateblue | 63 | salmon | 1456 |
| darkturquoise | 126 | salmon4 | 56 |
| floralwhite | 67 | sienna3 | 86 |
| greenyellow | 235 | skyblue | 106 |
| grey | 25 | skyblue1 | 98 |
| grey60 | 151 | skyblue2 | 43 |
| honeydew1 | 48 | skyblue3 | 85 |
| ivory | 69 | steelblue | 1702 |
| lavenderblush3 | 49 | tan | 228 |
| lightcyan | 164 | thistle2 | 61 |
| lightcyan1 | 72 | violet | 92 |
| lightgreen | 146 | white | 114 |
| lightpink4 | 49 | yellow | 778 |
| lightsteelblue1 | 73 | yellow4 | 38 |
| lightyellow | 134 | yellowgreen | 85 |
| magenta | 322 |  |  |
